# Chronotype and biological rhythms in bipolar disorders

**DOI:** 10.1192/j.eurpsy.2021.1653

**Published:** 2021-08-13

**Authors:** A. Ben Cheikh Ahmed, U. Ouali, I. Bouslama, Y. Zgueb, F. Nacef

**Affiliations:** 1 Department Psychiatry A, Razi Hospital, Manouba, Tunisia; 2 Department Psychiatry A, Razi University Hospital, La Manouba, Tunisia

**Keywords:** Bipolar Disorders, biological rhythms, chronotype, BRIAN scale

## Abstract

**Introduction:**

Biological rhythms play an important role in the etiology of mood disorders. Several lines of evidence established a link between circadian rhythm disruption and mood episodes. Chronotypes are the behavioral manifestations of circadian rhythms and eveningness appears to be more frequent in bipolar disorder (BD). The influence of chronotype on mood symptoms needs yet to be clarified.

**Objectives:**

-Identifying the predominant chronotype in a Tunisian sample of patients with BD -Assessing the association between chronotype and biological rhythm disruptions in the sample

**Methods:**

For this study, a total of 80 euthymic outpatients with bipolar disorder and 80 control subjects were recruited. Biological rhythms disruptions were assessed using the Biological Rhythm Interview of Assessment in Neuropsychiatry (BRIAN). Predominant chronotype was identified using the composite scale of morningness (CSM).

**Results:**

BRIAN scores showed greater biological rhythms disruptions in bipolar patients than the control subjects (mean scores 35.26±9.21 vs 25.84±2.68). Low CSM scores in the patients’ group indicated a predominant evening chronotype whereas an intermediate chronotype was more frequent within the control group. The correlation analysis revealed a statistically significant negative correlation between the 2 scales (r=-0.716, p<0.001): the CSM scores decreased as the BRIAN scores increased.

**Conclusions:**

This study indicates that eveningness is more common in BD. This chronotype is more likely to disturb biological rhythms which may increase the risk of mood symptoms and lead to a poor prognosis for BD, thus the relevance of treating rhythm alterations, especially in evening-type patients, in order to improve their quality of life and prevent mood episodes.

**Disclosure:**

No significant relationships.

